# A deep-learned skin sensor decoding the epicentral human motions

**DOI:** 10.1038/s41467-020-16040-y

**Published:** 2020-05-01

**Authors:** Kyun Kyu Kim, InHo Ha, Min Kim, Joonhwa Choi, Phillip Won, Sungho Jo, Seung Hwan Ko

**Affiliations:** 1 Applied Nano and Thermal Science Lab, Department of Mechanical Engineering, Seoul National University, 1 Gwanak-ro, Gwanak-gu, Seoul, 151-742 Korea; 2 Neuro-Machine Augmented Intelligence Laboratory, School of Computing, Korea Advanced Institute of Science and Technology (KAIST), Daejeon, Korea; 3 Institute of Advanced Machinery and Design (SNU-IAMD)/Institute of Engineering Research, Seoul National University, Seoul, 08826 Korea; 4 Laser Thermal Lab, Department of Mechanical Engineering, University of California, Berkeley, CA 94720 USA

**Keywords:** Sensors and probes, Mechanical engineering, Materials for devices, Sensors

## Abstract

State monitoring of the complex system needs a large number of sensors. Especially, studies in soft electronics aim to attain complete measurement of the body, mapping various stimulations like temperature, electrophysiological signals, and mechanical strains. However, conventional approach requires many sensor networks that cover the entire curvilinear surfaces of the target area. We introduce a new measuring system, a novel electronic skin integrated with a deep neural network that captures dynamic motions from a distance without creating a sensor network. The device detects minute deformations from the unique laser-induced crack structures. A single skin sensor decodes the complex motion of five finger motions in real-time, and the rapid situation learning (RSL) ensures stable operation regardless of its position on the wrist. The sensor is also capable of extracting gait motions from pelvis. This technology is expected to provide a turning point in health-monitoring, motion tracking, and soft robotics.

## Abstract (web summary)

Real-time monitoring human motions normally demands connecting a large number of sensors in a complicated network. To make it simpler, Kim et al. decode the motion of fingers using a flexible sensor attached on wrist that measures skin deformation with the help of a deep-learning architecture.

## Introduction

For exploring and understanding complex systems like the earth and the universe, monitoring its different parts is essential. This can be achieved by obtaining the status of each part through various signals such as radio waves, mechanical vibrations, and electricity. Since these signals are generated by many components that are widely distributed throughout the systems, their acquisition and integration are key to understanding their nature. Advanced technologies, like the global seismographic network and the radio telescope system, facilitate the collection of signals by placing highly sensitive detectors at positions where signals converge. This enables the decoupling and integration of the information entangled in the converging signals into one frame of knowledge for the observation of the entire system.

A similar approach can be applied to monitor the complex movements of the human body. Current methods that directly measure joint^1–7^ and muscle activity by electromyography (EMG) signals^8–10^ embody inefficiencies since detecting signals from each joint requires a great number of sensors connected with thousands of outer wirings in order to decode human movements. Furthermore, EMG signals are not only affected by inconsistencies due to the coupling between neighboring muscles but also require a large number of sensors^11–13^, introducing time and labor-intensive data preprocessing. Such impracticality can be avoided by developing and using a suitable, highly sensitive sensor rather than pinpointing every joint and muscle of our body.

We therefore propose an ultrasensitive skin-like sensor that measures previously undetectable signals from small skin deformations that are far from the joint, coupled with a deep neural network that clarifies the movement of the corresponding body parts. The sensor is attached onto the wrist and is capable of extracting signals corresponding to multiple finger motions, providing a method to understand the human body that is more efficient than pinpointing every joint and muscle. Laser-induced nanoscale cracking allows for the sensor to achieve high sensitivity, and the performance of the sensor was engineered through a concrete theoretical model. A consecutive laser serpentine patterning also allows for the sensor to conformably attach to the epidermis. The deep neural network successfully decodes the temporal sensor signals from the wrist to generate the corresponding finger motion. Our RSL system guides users to collect data from arbitrary part of the wrist and automatically trains the model in real-time demonstration with a virtual 3D hand that mirrors the original motions. The sensor is also applicable to the pelvis and can successfully generate dynamic gait motions in real-time. These would facilitate an in-direct remote measurement of human motions which can be used in various applications that rely on motion perception such as rehabilitation, prosthetics, human–machine interfaces, and wearable haptics for virtual reality.

## Results

### Deep-learned skin-like sensor system

**Fig. 1:**
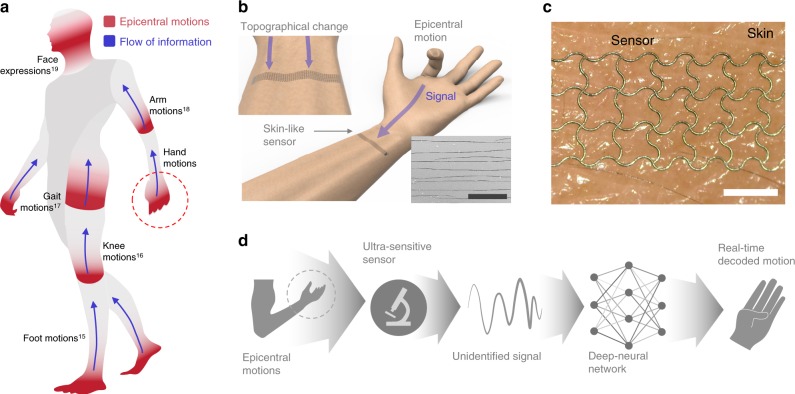
Design of the deep-learned skin-like sensor. **a** Schematic depicting the possible flow of information through our body. The information may include foot, knee, hand, arm, gait, and also face expressions. **b** Illustration of measuring the epicentral motions of fingers. Upper left image depicts the measurement of the topographical change of the wrist caused by the finger motions. Lower right image shows the SEM image of the cracked region of the sensor. Scale bar, 40 μm. **c** Magnified image of the sensor conformably attached on skin. Scale bar 1 mm. **d** Design of the proposed sensory system.

An illustration of motions in human body is shown in Fig. 1a. Movement of any joint is associated to its surroundings^14^, involving electrical signals such as action potential of muscle, or mechanical signals of skin deformation. The blue arrows highlight the likely information flow caused by the movement from the main joints. Attempts to capture these signals are numerous including measuring the movement of the foot from shin^15^, knee movements from thigh^16^, and information converging around the pelvis^17^ with signals representing the entire gait motions. Similarly, motion of the arm^18^ and the face expression^19^ can be also identified. Predicting the status of motion aside from the main joints is like earthquake prediction, mainly involving time, location, and magnitude. Similarly, the aim of our study is to decode and extract the “epicentral” motions from the detected signal. Among the numerous motions generated in the human body, hand exhibits the highest degree of freedom which exquisitely performs a range of tasks^20^; hence, predicting its motions is very challenging. Our study, therefore, initially focused on decoding the dynamic finger motions in real-time (Supplementary Table 1). Figure 1b illustrates the platform of the sensing system. A topographical movement of the wrist is triggered by the epicentral finger motion, with the attached crack-based sensor producing a signal containing the motion information. A sample scanned electron microscopy (SEM) image of the sensor crack is shown in the lower right corner. The magnified image of the sensor attached above the skin is shown in Fig. 1c. The serpentine patterns allow a conformal contact with the epidermis, enabling a more direct measurement of skin deformation. The design of our analysis is shown in Fig. 1d. The wrist contains information reflective of several finger motions. The highly sensitive crack-based sensor detects the deformation of the wrist as unidentified signals. The signals are then analyzed in a temporal sequence through our encoding network, and the current status of the motion is simultaneously generated through the decoding network.

### Highly sensitive sensor by laser-induced crack generation

**Fig. 2:**
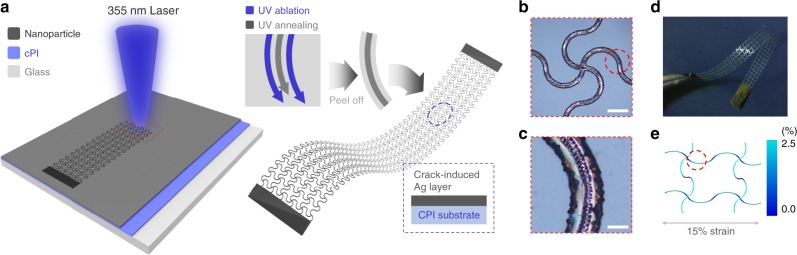
Highly sensitive skin sensor fabrication by laser-induced crack generation. **a** Schematic depicting the patterning and crack fabrication by laser fabrication. **b** Optical image of the fabricated sensor. Scale bar, 200 μm. **c** Magnified image of the sensor which distinctively shows the annealed region. Scale bar, 50 μm. **d** Picture of the free-standing fabricated sensor. **e** FEM image showing strain distribution of the sensor.

The process requires a sensor that is sensitive enough to measure the minute deformation while holding high conformability with the skin in order to catch the subtle topology transitions of the wrist. Digital laser fabrication provides a viable solution to obtain both features through laser controlled cracking and serpentine patterning (Supplementary Fig. 6a, Supplementary Table 2). The periodic serpentine structure exhibits higher level of elastic deformation^21^, causing a conformal contact between the electrode and skin^22^; this promotes sensing of minute skin deformation. A crack-induced layer with micro serpentine patterns can easily be generated by simply scanning the laser with different power conditions. Cracked layer is used as a sensing element, since these structures are widely utilized^23–25^ in detection of minute mechanical stimulations. Figure 2a illustrates the fabrication process and the structure of the sensor. Colorless polyimide (CPI) is uniformly coated on a glass substrate and fabricated silver nanoparticle (AgNP) ink is then spin-coated over the layer. The bilayer of AgNP and PI is firstly patterned into the serpentine structure through a 355 nm wavelength laser ablation (over 100 mW). This process^26–28^ is better than the conventional fabrication method^29,30^ often requiring high temperature, vacuum environment, or a preprocessed mold. Subsequently, the laser power is lowered within a certain range (6–13 mW) to selectively convert the AgNPs into a crack-induced layer. The patterned structure is easily peeled from the glass substrate, with Fig. 2b, c depicting the magnified optical image of the final structure. The sensor performance is controlled through the annealing region as depicted in the middle line of Fig. 2c. The fabricated free-standing sensor is displayed in Fig. 2d.

The sensor is directly mounted on the skin through the assistance of adhesive PDMS. The strain distribution of the sensor under 15 % strain is observed through a finite element method (FEM, COMSOL Multiphysics) as illustrated in Fig. 2e. On account of the out-of-plane buckling deformation of the sensor, an effective strain under 2% is applied through the electrode. The performance of the sensor under the serpentine pattern is discussed in Supplementary Note 5, Supplementary Fig. 5.

### Correlation between cracking and control parameters

Previous works on crack-based ultrasensitive sensors mainly involve fabrication by bending a metal-sputtered soft substrate^23,24,31,32^. The performance of the conventional sensors is engineered through varying substrate thicknesses, substrate modulus, annealing times, and using stress concentration structures^23,25,32,33^. However, previous approaches failed in deducing the relation between the control parameters and the sensor performance. The conventional technology barely explains the grain size that is associated with crack characteristics. A correlation between these can be examined by analyzing the signal outputs during the initial crack formation, whereas previous studies relied on the data obtained from the sample after cracking ends.

**Fig. 3:**
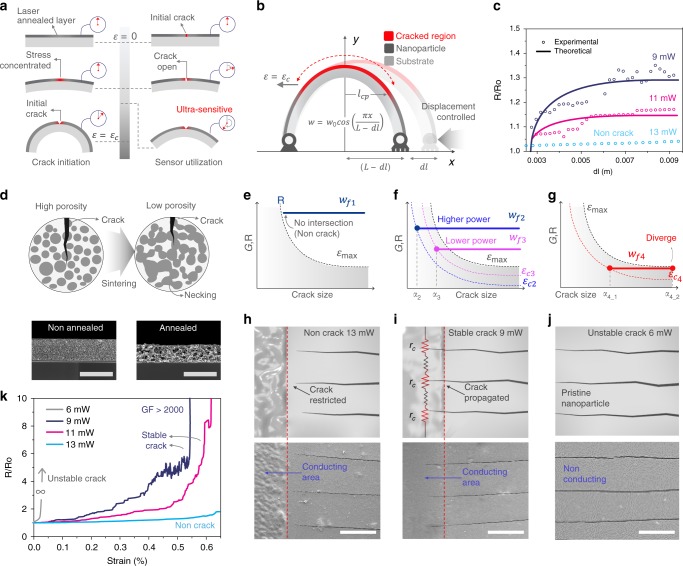
Investigation of crack characteristics. **a** Electrical response while initial cracking (left) and sensor operation (right). **b** Schematic illustration and modeling parameters of displacement controlled bending environment. **c** The initial cracking resistance changes of the sensors prepared by different laser power with model-fitting curve. **d** Schematic representation of sintered particle layer and SEM image of the cross section of annealed (right) and non-sintered (left) particle layer. Scale bar, 40 μm. **e**–**g** Energy release rate curve and resistance function of “non-cracking” (**e**), “stable cracking” (**f**), and “unstable cracking” (**g**). Arrow at the bottom describes the increasing direction of electrical conductivity (blue) and degree of crack (gray). **h**–**j** Crack appearance for each regime, “non-cracking” (**h)**, “stable cracking” (**i**), and “unstable cracking” (**j**). The first row indicates the schematic of crack length and the second shows the SEM images respect to each case. Scale bar, 14 μm. **k** The operating output resistance of the sensors prepared by different laser power.

As depicted in Fig. 3a, initial cracking of the laser annealed layer is proceeded before utilizing as a sensor, and the electrical response in respect to strain in the cracking process significantly varies from that in the sensor operation process. The electrical resistance discontinuously increases in the initial micro cracking, whereas in the sensor utilization, a continuous change of resistance is observed since the gap of the crack is widened continuously. The discontinuous nature of initial cracking makes it difficult to obtain meaningful information from the signal output; therefore, we designed a bending test under quasi-static conditions as shown in Fig. 3b, leading the set of discontinuous cracks to propagate continuously along the line. Crack occurs in regions where the local strain *ɛ* is higher than the critical crack strain *ɛ*_*c*_ (red in Fig. 3b), and do not occur below the critical strain (black in Fig. 3b). Since the first buckling mode of thin film is defined as a sinusoidal form, the curvature of the deformed sensor is represented as cosine curve (Supplementary Note 1, Supplementary Fig. 1).

The projected length of cracked zone *l*_cp_ is defined implicitly as follows:

1 $${\it{\upvarepsilon }}\left( {{\it{l}}_{{\mathrm{cp}}},{\it{dl}}} \right) \,=\, {\it{\upvarepsilon }}_{\it{c}},$$

where *ɛ* is the local strain of the sensor and *dl* is the displacement of the bending stage. The resistance ratio between the non-cracked and cracked region is defined as *α* = *r*_*c*_/*r*_*n*_ where *r*_*c*_ is the resistance per unit length of the cracked zone and *r*_*n*_ is the resistance per unit length of the non-cracked zone. The normalized resistance of the sensor according to the displacement *dl* of the bending stage is expressed as:

2 $$\frac{{R(dl)}}{{R_0}} \,=\, \frac{2}{L}\left( {\alpha \,-\, 1} \right)l_{\mathrm{c}}\left( {dl} \right) \,+\, 1,$$

where *R*_0_ is the initial resistance of the sensor and *l*_*c*_ is the length of cracked zone. The detailed derivation of Eqs. 1 and 2 are found in Supplementary Note 1. In this model, two free factors, *ɛ*_*c*_ and *α* that determine the final shape of the electrical response are found by fitting the experimental data as shown in Fig. 3c. Higher laser power decreases the *ɛ*_*c*_ and *α*; *ɛ*_*c*_ = 2.977 × 10^−4^,*α* = 1.846 for 9 mW, *ɛ*_*c*_ = 2.4 × 10^−4^,*α* = 1.4 for 11 mW. Conditions under 6 mW are not enough to provide electrical pass ways through annealing between the particles and cases above 13 mW cannot be appropriately fitted by two free factors (Supplementary Fig. 6b).

Selective laser sintering provides a sophisticated method for manipulating the critical crack strain. As illustrated in Fig. 3d, high power annealing lowers the porosity of the particle layer and provides a higher bonding energy per unit area, *w*_f_ through the necking between particles^34,35^. According to Irwin^36^, a crack propagates further when the following condition is satisfied.

3 $$G_{\mathrm{c}} \,=\, - \frac{{dU}}{{dA}} \,=\, R,$$

where *G*_*c*_ is the critical energy release rate, *U* is the potential energy of body, *A* is the crack area, and *R* is the resistance function. The typical energy release rate for the displacement-controlled case gradually decreases with the crack size^37^ as depicted in Fig. 3e–g. The resistance function, represented by the right-hand side of Eq. 3 is defined as follows:

4 $$R\left( {{a}} \right) = 2w_{\mathrm{f}}H(a - a_0),$$

where *a* is crack size, *H*(*x*) is the Heaviside step function, and *a*_0_ is the void size determined by the porosity of the sintered layer. Since critical cracking occurs at the intersection point of the *G* and *R* curve interpreting Eq. 3, the resistance function is categorized into three cases based on the relative position of *G* to the maximum strain *ɛ*_max_; these include non-cracking (Fig. 3e), stable cracking (Fig. 3f), and unstable cracking (Fig. 3g).

The excessive power of the laser anneals the particle layer into a fine bulk metal structure with high bonding energy. Since the *R* curve is above the set of *G* curves in Fig. 3e, the intersection point is inexistent; thus, the crack cannot propagate further and maintains its initial size. We found that the condition for non-cracking was above ~13 mW. The illustration in Fig. 3h and the SEM image demonstrate that the crack is restricted at the boundary of the annealed area, restricting cracking of the sensor’s active area (conducting area). Meanwhile, at low power annealing condition under 6 mW provides an inadequate bonding energy to bypass the envelope of *G* curves as shown in Fig. 3g. In such a case, the *R* curve with bonding energy *w*_f4_ meets the *G* curve corresponding to some strain *ɛ*_c4_; however, the equilibrium crack size is infinitely large since the intersection point with maximum strain *ɛ*_max_ diverges. Moreover, the particle layer lacks the electrical path delivering sensor signals due to insufficient annealing power, and the corresponding crack feature is depicted in Fig. 3j. The annealing condition between unstable cracking and non-cracking involves a distinct intersection point of the *G* and *R* curves throughout the straining range as shown in Fig. 3f, with a finite equilibrium crack size for various conditions (stable cracking). In this condition, easy manipulating of the critical crack strain of the resultant structure is possible by varying the laser power. For instance, the higher power induces a smaller void size (*a*_2_) and higher bonding energy (*w*_f2_) on the structure, causing a smaller critical crack strain (*ɛ*_c2_ < *ɛ*_c3_). We already confirmed such a relation in the interpretation in Fig. 3c. To investigate the dependence of the annealing power on the sensitivity in the stable crack regime, we found the correlation between the critical crack strain and the length of crack is given by:

5 $$\varepsilon _c^2\,\sim\, \frac{{4b}}{{L^2}}p,$$

where *b* is the thickness of the sensor, *L* is the length of the sensor, and *p* is the propagated length of the crack (Supplementary Note 2). Equation 5 shows that the square of the critical crack strain is proportional to the propagated length of the crack. As shown in Fig. 3i, the crack is propagated with a certain crack length and reduces the conducting path of the sensor, which in turn, increases the resistance ratio *α*. Moreover, *p* directly represents the grain structure of the sintered area, whereas other properties like young’s modulus are combined with other physical properties to define the grain size. A structure with larger grain size yields longer *p* and smaller crack asperity since the formation of the crack scatters less at the coarse grain boundary^38^. Moreover, the distribution of the crack asperity exhibits fractal similarity to the grain size distribution by the renormalization theory. Since the finely cracked face responds sensitively under strain ( Supplementary Notes 3, 4, Supplementary Figs. 2 and 4), the sensor with larger *p* is more sensitive (Gauge Factor (GF) > 2000 at 0.55%) as shown in Fig. 3k. More detailed information on the validity and the relationship between Eq. 5 and the fitted *ɛ*_c_ and *α* is discussed in Supplementary Note 2*.* The overall process of the theoretical analysis is summarized in Supplementary Fig. 7.

### Learning the dynamic motions with a single sensor

**Fig. 4:**
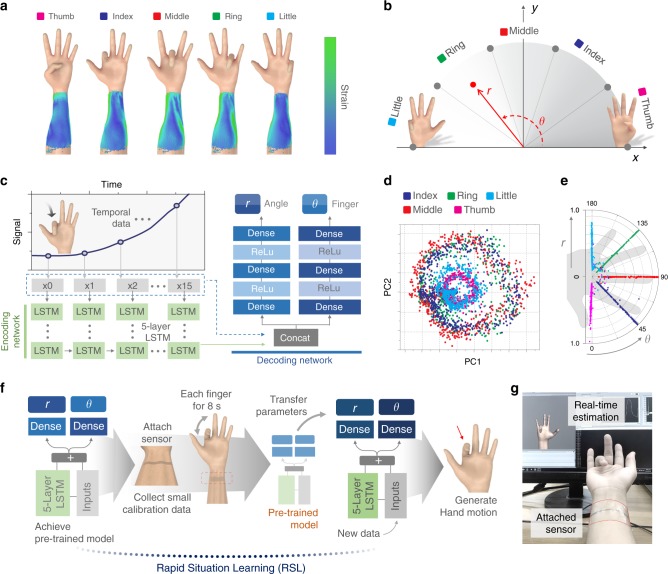
Identifying human hand motions from detected skin deformation. **a** Depiction of skin deformations for different finger bending motions. **b** Metric space defining single finger bending motions: physical alignment of fingers in a hand is expressed in the metric space with *R* representing the amount of a finger bent and *θ* identifying the position of a finger in a hand. **c** Neural network is composed of an encoding network and a decoding network. LSTM layers are used in encoding network to analyze temporal sensor patterns to generate latent vectors. Two independent dense layers map created latent vectors to our metric space expressing hand motions. Dropout is used as the regularization technique to prevent the network to be overfitted to a single use case. **d** 2D PCA illustration of output vectors produced by encoding network. Each circular cluster demonstrates that encoding network can correctly identify cyclic finger motions from sequential sensor inputs. **e** Figure of how sensor inputs in training dataset are mapped to the metric space after passing our network. **f** The processes of rapid situation learning (RSL) that utilizes transfer learning. When the sensor is attached to a new position and a small amount of retraining data is collected, the new network utilizes knowledge learned during pretraining by transferring parameters from pretrained network, reducing the amount of dataset, and time for retraining. **g** Photo of actual hand motion generation.

We used a deep neural network to identify complex hand motion from highly sensitive sensor signals. As illustrated in Fig. 4a, various hand motions result in signals from skin deformations and muscle movements. To guide our network to correctly identify the moving finger, we defined a metric space as in Fig. 4b. The *R* values express the bend of a finger while *θ* values represent the identity of the moving finger.

The metric is designed to consider the spatial positions of the fingers and how humans distinguish different hand motions. It is much harder to distinguish hand motions when the fingers are barely bent than when they are fully bent. Furthermore, the motions of two fingers apart are more easily distinguished than motions of two fingers that are close to each other. Therefore, to represent this, points on our metric space are closer to each other when *r* and the difference between their *θ* values are lower. This Euclidean distance between points is used as our network’s loss function to help it learn to differentiate different hand motions. For example, if the little finger is the finger that is bent, we pose a higher penalty for our model when it incorrectly determines the bent finger as the thumb than when it incorrectly determines it as the ring finger.

Therefore, we designed neural network to accomplish two tasks: firstly, analyzing sensor signal patterns into a latent space encapsulating temporal sensor behavior and secondly, mapping latent vectors to our finger motion metric space defined above. Encoding and decoding network in Fig. 4c achieve above goals respectively. To maximize user convenience regarding usability and mobility^39,40^, we used a single-channeled sensor to generate signals corresponding to complex hand motions. Thus, it was necessary to utilize temporal sensor patterns to correctly determine the hand motion the signals were generated from. We therefore trained a long short-term memory (LSTM) network^41^, a type of RNN architecture, to identify such temporal behaviors (Fig. 4c), as it is a type of deep neural network designed to analyze sequential data. Detailed description of the LSTM is included in Supplementary Note 6. To map latent vectors into corresponding points in our 2d metric space, the decoding network is composed of two separate dense layers, mapping encoded latent vectors into *r* and *θ* respectively.

The resulting vectors from our network are visualized in Fig. 4d. We used principal component analysis to project the latent vectors onto the 2D vector space. In general, the sensor signals corresponding to a specific finger create a circle in the 2D vector space. Since the finger motions involve a cycle of bending and unbending the finger between a starting straightened position and an ending bent position, this observation is expected. However, there are two main changes to the data after it is passed through the encoding network (Supplementary Fig. 10). Firstly, the starting points, where all fingers are straightened, are aligned by the encoding network. By labeling the input vectors as a point in the half-circle metric space that we defined, we intended to represent the starting points as closer vectors in our metric space. The alignment above demonstrates that our model maps straightened finger motions to closer latent vectors as we intended. Secondly, the data points for the ring finger, which were widely distributed across the projected 2D plane before encoding, create a circle with a radius similar to those of the other fingers after encoding. The encoding network transforms different data points to latent vectors that represent their corresponding finger motion. Therefore, even if the original sensor signals had different values, they are still projected to similar latent variables as long as they correspond to the same finger motion. This demonstrates that our network correctly utilizes temporal sensor behavior to analyze the different patterns for each finger motion. Figure 4e shows the generation of *r* and *θ* values by the dense layers from the network-produced vectors mapped to the metric space. Even though some data points are misclassified when the *r* value is low, dense layers clearly discriminate different finger motions when the fingers are significantly bent and the *r* values are high. A real-time demo of our network analyzing sensor signals from the hand motions of the sensor wearer can be seen in Fig. 4g and the Supplementary Movie 1.

Finger motions are generated by analyzing the strain changes at the subject’s wrist site. However, a simple wrist movement can also modify sensor signals by producing non-finger motion noises. To verify whether our sensor can generate signals that allow our model to distinguish between different noises and finger motions, we conducted an additional experiment to check if our model can classify five motions and three types of noises generated by non-finger bending motions as shown in Supplementary Fig. 11a. Three noises are sensor signals caused by directly touching the sensor, twisting the wrist, and bending the wrist, we call them touch, twist, and wrist respectively.

To perform the classification task, we modified the decoding network to a three-layered dense block producing 8-dimensional vector output. Each value in an output vector is model predicted probability for each eight classes. A class with a maximum probability is chosen as the model predicted class for a given sensor input. As illustrated in the confusion matrix in Supplementary Fig. 11b, our model could correctly classify finger motions and noises with 96.2% in average and 92.9% in the worst case for little finger motions. The result shows that our sensor can generate distinctive signal patterns for different hand motions including non-finger motions so that our model can distinguish finger motions from noises generated by three non-finger motions.

From the above results, we know that given the sensor data of a user, our network is trained to correctly classify the user’s finger motions. However, attaching the sensor to a different user, the muscle movements and sensor values corresponding to the hand motions of the new user may be different from those of the previous users, as human muscle movement vary from person to person. Since our network is trained for different sensor patterned dataset, the network may consequently fail to determine hand motions by the new user. We therefore need to retrain the network with the new data from the new user. However, if we train our model from scratch, we need at least 2000 sensor frame from 80 s of finger movement for each finger. It is impractical and inconvenient to collect a 400 s of training dataset each time the sensor is attached to a new user. Even if we were to collect enough data, the training time necessary for the LSTM network to extract the hidden sensor patterns from the dataset is too high. Similar issues arise when the sensor is attached to an area different from before or when the sensor itself is replaced with a new one. These problems hinder practical applications aimed for usage by multiple end users.

To address these problems, we designed the RSL, a deep-learning system guides user to collect data and automatically processes them to retrain our models with only a small amount of data in a short period of time. The procedure of the system (Fig. 4f) involves following onscreen instructions to collect data for 8 s per finger when the sensor is placed on a new user (Supplementary Fig. 12). By sliding a time window of size 16, we group the collected data to form 16 consecutive sensor signal input. The generated input is used as a single input for our model. The detailed data processing is described in Supplementary Note 6.

The RSL system uses transfer learning^42^ techniques to utilize knowledge on sensor behaviors obtained during previous training steps. The parameters for the LSTM and dense layers are then transferred from the pretrained model to the new model. After retraining for around 5 min with the newly collected data, the model is then ready to generate the hand motions of the new user.

Through RSL system, all steps required for generating the hand motions of a new user are processed automatically. Typically, the temporal behavior patterns of the sensor signals that were already previously analyzed by our pretrained model is transmitted to the new network. Consequently, the retraining time is massively reduced because the network only needs to retrain its mapping functions to map input values to a different range of sensor values. The effectiveness of using transfer learning is evident in the loss comparison graph (Supplementary Fig. 8). In the absence of transfer learning, over 20 min are required for the loss to decrease to below 0.1, whereas in its presence, the time is within 5 min for the same dataset. The detailed information regarding the network design can be found in Supplementary Note 6.

As a proof-of-concept demonstration of our system’s expandability, the sensor is used to decode the keyboard typing of numpad which the signals are combined with the movements of the wrist and the finger. The modified model decoded 9 classes of number in real-time that are pressed by fingers (Supplementary Note 7, Supplementary Fig. 13). Moreover, a single sensor is also attached on pelvis to identify the gait motions. The modified model (Supplementary Note 8) successfully generated the positions of the ankle and knee as shown in Supplementary Figs. 14, 15. Moreover, the signals are collected in the cases where the wrist and the finger movements are coupled.

## Discussion

Inspired by the understanding of detection techniques for measuring converging signals, we present a technique for measuring dynamic motions by a deep-learned soft sensor attached on the surface of the skin, that is, superior to conventional approaches. Apart from the traditional wafer-based fabrication, the proposed laser fabrication provides a powerful solution for viable sensor utilization. The relationship between the sensor performance and the controlling parameters was investigated to ensure precise manipulation. A deep neural network is synchronized with the measuring equipment and the sensor, demonstrating a perfect operation in decoding finger motions. The concept of our system is expandable to other body parts, and offers great potential for detecting other stimuli and physiological signals. For device expansion on other body parts, a concrete ergonomic analysis will be needed to select an optimum location to measure epicentral motions. Methods of selecting required number of sensors and technique of integrating with wireless platform is necessary for practical use.

## Methods

### Synthesis of silver nanoparticle ink

0.25 mol/l of silver nitrate (99% Aldrich) was used as a precursor and dissolved in ethylene glycol (EG, 99.9%, Aldrich) with 0.02 mol/l of polyvinylpyrrolidone (PVP, Mw = 10,000). The solution was stirred in a reaction condition of 150 °C until the synthesis was completed. The synthesized particles were then separated by centrifugation of 7000 rpm for 30 min and washed by ethanol. The collected particles were re-dispersed in ethanol at a concentration of 30 wt%.

### Data acquisition and communication

Pretraining and recalibration data were received by digital multimeter (Keithley 7510, Keithley) at 40 hz of sampling rate. For the real-time demo, the sensor signals are recorded at an identical sampling rate, and simultaneously delivered to the learning network.

### Quasi-static bending test

Bending was applied by linear displacement (VT-80, PI) at a speed of 0.05 mm/s. The electrical signals were received at the same time in order to identify the relation between annealed nanoparticle layer and the critical cracking strain.

### Strain mapping of the wrist

Deformation of the wrist caused by the finger motions is measured by digital image correlation (DIC). A random speckle patterns were distributed over the wrist in order to process deformation mapping through DIC.

### Sensor attachment on skin

In order to conformably attach the electronics, the entire sensor is embedded with an adhesive PDMS, tuned by ethoxylated polyethylenimine (PEIE)^43^ as shown in Supplementary Fig. 3a. Magnified image of the attached sensor is depicted in Supplementary Fig. 3b. The vertical height information of the system is illustrated as Supplementary Fig. 3c.

### Experimental setup for direct laser writing

The optical system is consisted of pulsed 355 nm laser (Nanio Air 355-3-V, InnoLas Photonics). Synthesized AgNP ink (20 wt%) is spin coated on CPI substrate with 200 rpm and 1 min condition, which allows fine deposition and evaporation of the solvent.

## Supplementary information

Supplementary Information

Description of Additional Supplementary Files

Supplementary Movie 1

## Data Availability

The data that support the findings of this study are available from the corresponding authors upon request.
