# Supplementary material for: A deep-learned skin sensor decoding the epicentral human motions
Source: Nat Commun. 2020 May 1;11:2149. doi: 10.1038/s41467-020-16040-y (PMC7195472; doi:10.1038/s41467-020-16040-y)
Supplement: Supplementary file 1 — Supplementary Information [file 41467_2020_16040_MOESM1_ESM.pdf]

Supplementary Information

A deep-learned skin sensor decoding the epicentral human motions

Kim et al.

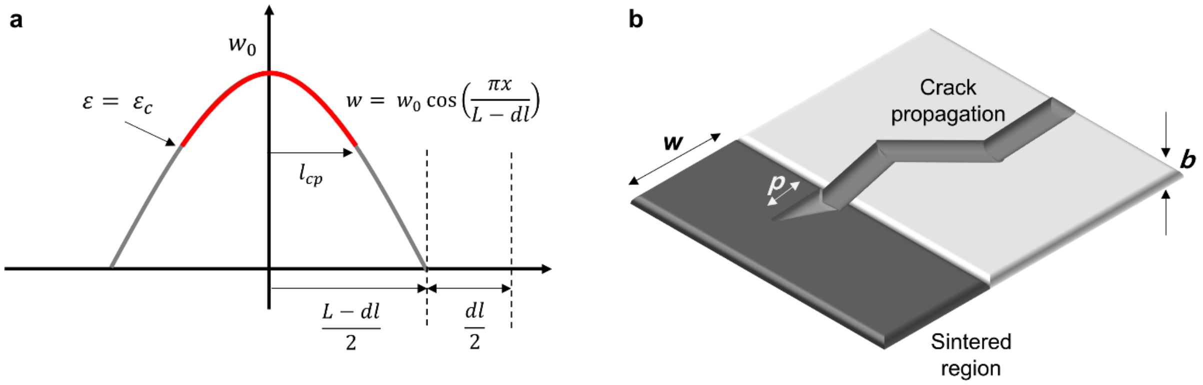

Supplementary Figure 1 | The modeling parameters related to **a.** the geometrical model and **b.** the thin film cracking model

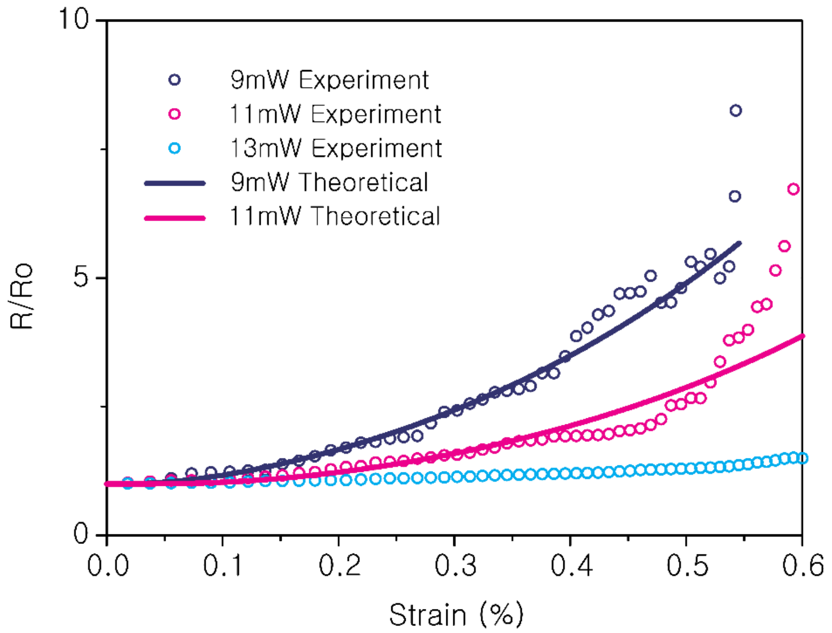

Supplementary Figure 2 | Optical property of lower layer. Absorbance at 532nm wavelength was calculated using reflectance and transmittance.

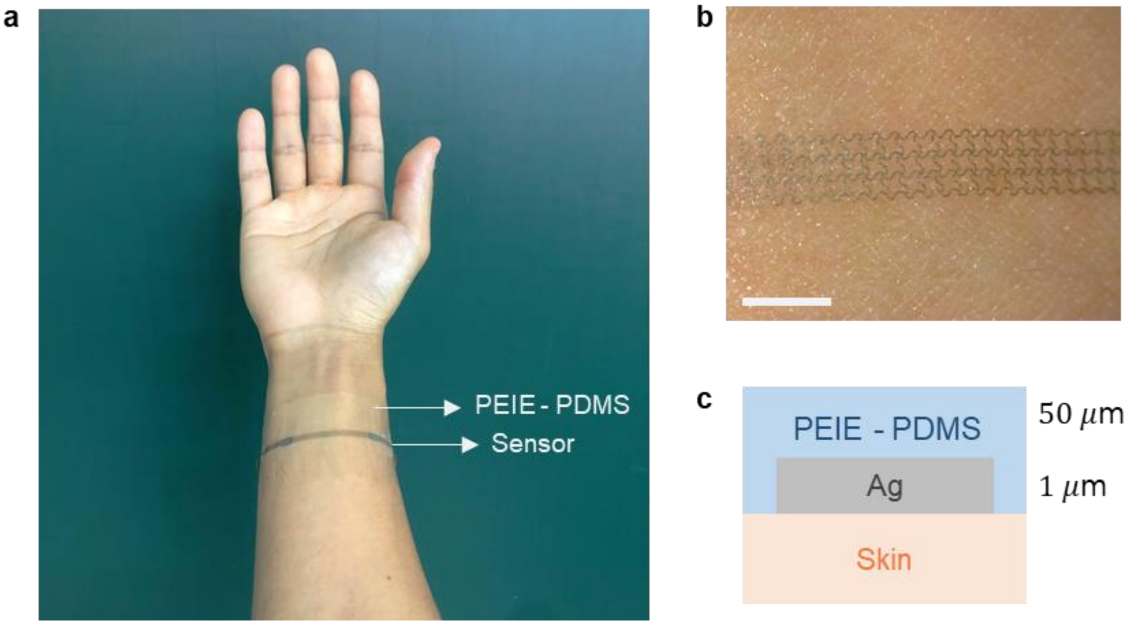

Supplementary Figure 3 | Attaching sensor with the assistance of elastomer **a.** Photo of the attached sensor. **b.** Magnified image of the attached sensor. **c.** Cross-sectional image of the attached sensor

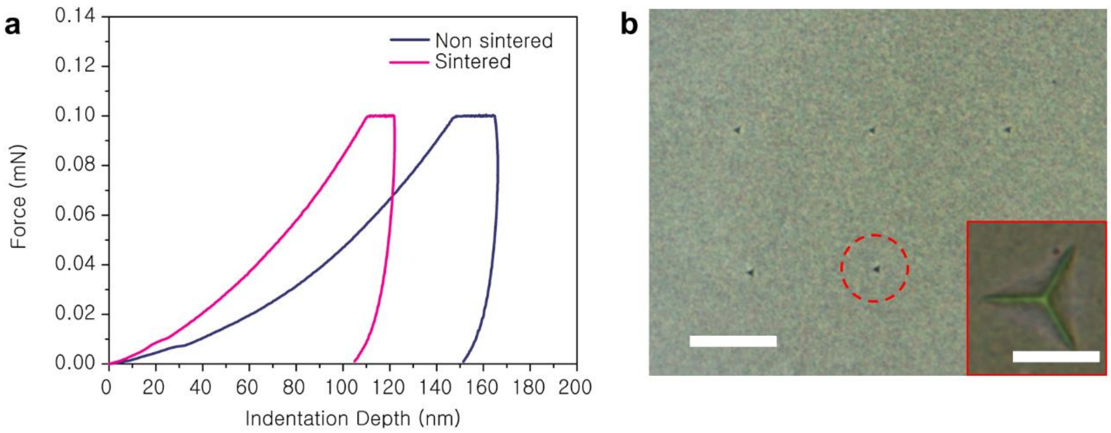

Supplementary Figure 4 | Nanoindentation test of the particle layer. **a.** Nanoindentation curves for pristine and sintered particles **b.** Indentation shape generated above pristine nanoparticle layer. Scale bars, 10 μm and 2 μm (inset)

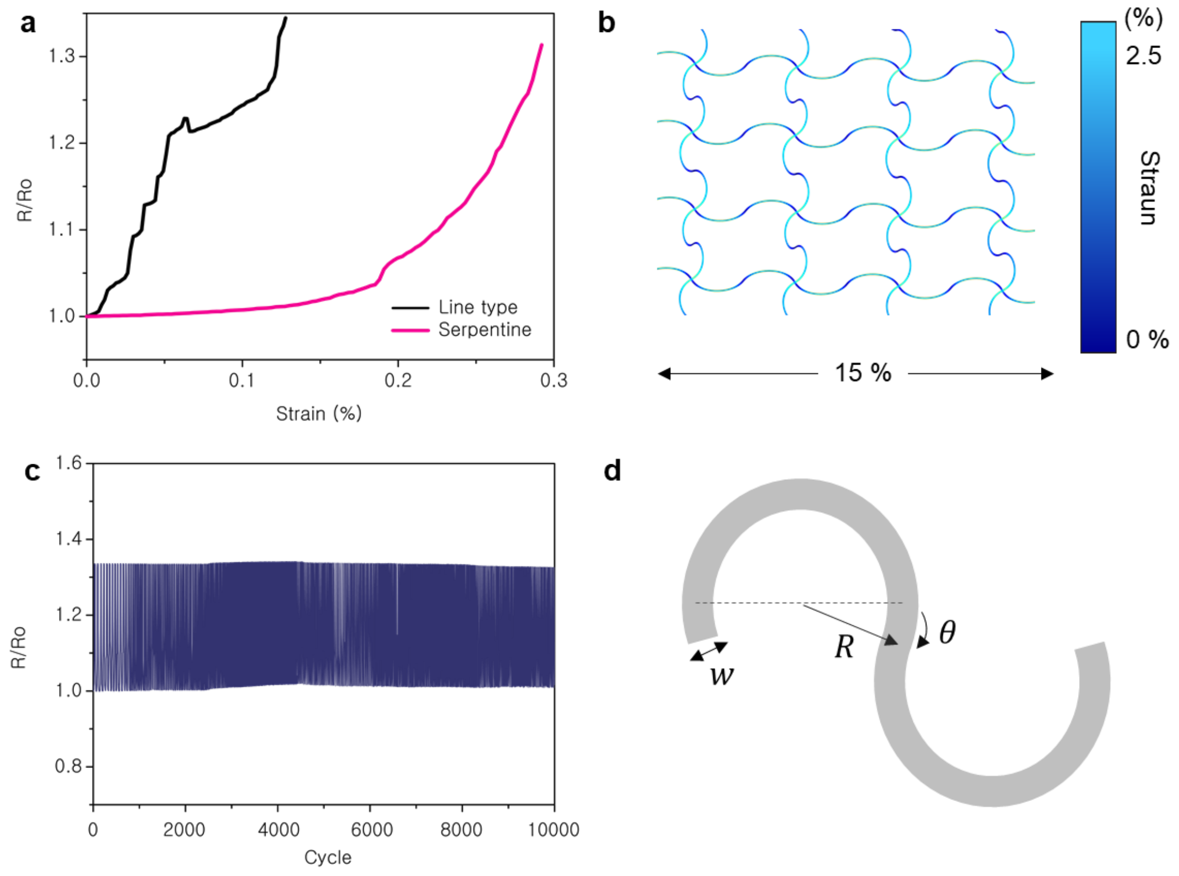

Supplementary Figure 5 | Mechanical properties of the patterned structure. **a.** Sensitivity difference between non-patterned structure and serpentine structure. **b**. FEM simulation of the sensor with strain under 15%. **c.** Electrical stability under stretching in 0.3% strain. **d.** Shape parameter of the serpentine structure. R=200 µm, w=30µm.

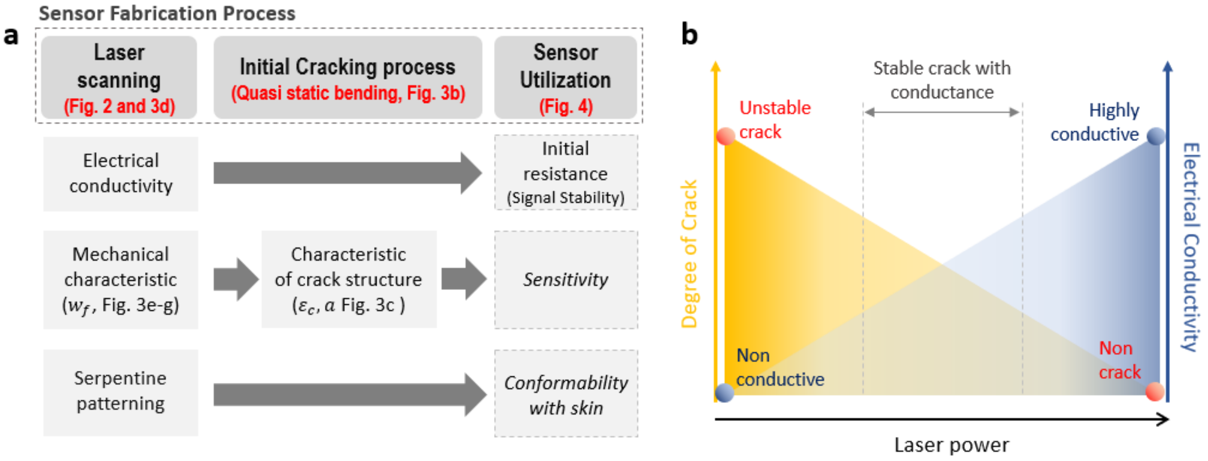

Supplementary Figure 6 | Main performance indicators during the fabrication process. a. Influence of laser condition on the parameters. **b.** Relation between laser power and the parameters.

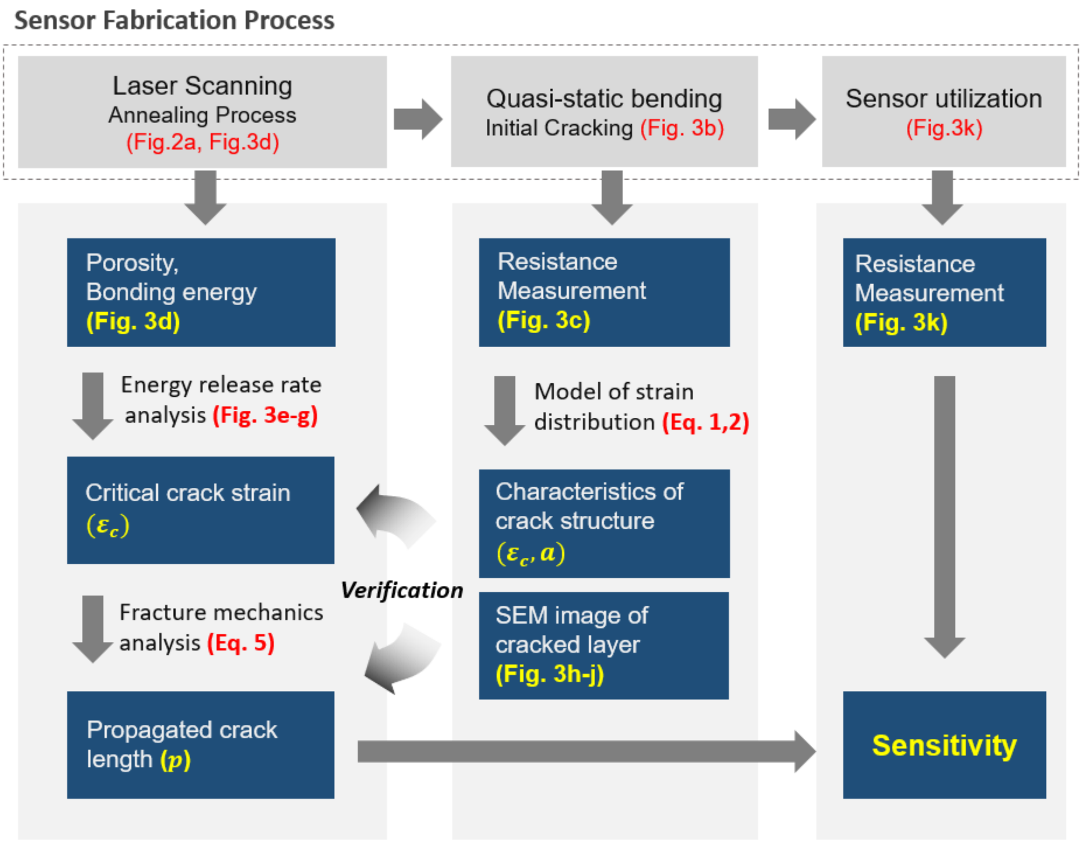

Supplementary Figure 7 | Theoretical analysis defining sensor's sensitivity

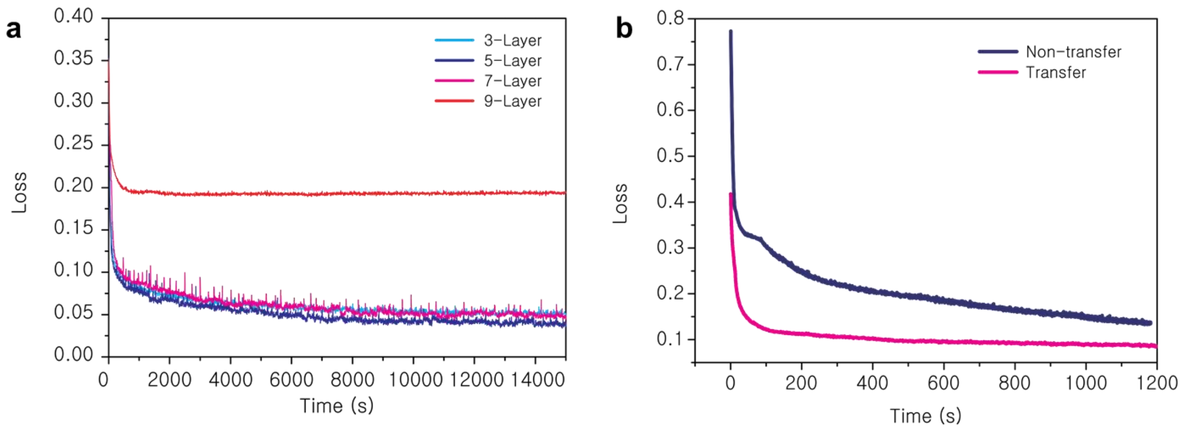

Supplementary Figure 8 | Learning characteristics. **a.** Varying LSTM layers and loss difference. **b**. Loss difference between non-transfer and transfer learning data.

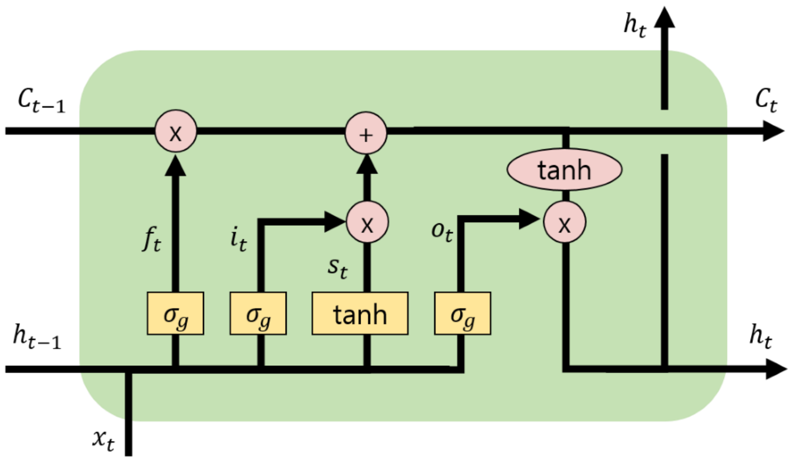

Supplementary Figure 9 | Structure of the LSTM unit

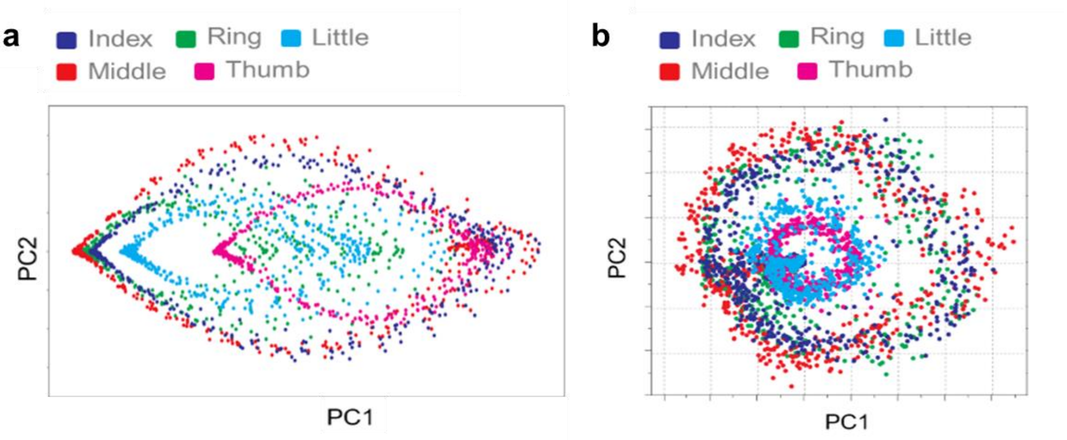

Supplementary Figure 10 | PCA analysis of sensor a. PCA before passing encoding network. **b.** PCA after passing encoding networks.

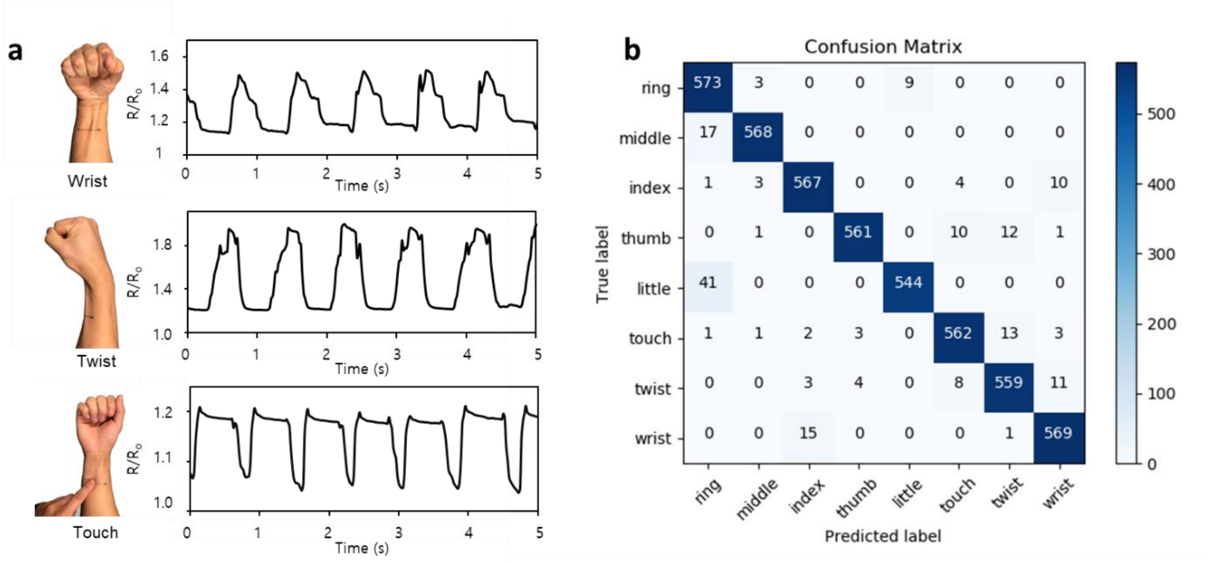

Supplementary Figure 11 | Noise analysis of the sensor a. Signal outputs of various noise. **b.** Confusion matrix of decoding finger motions included with external noise.

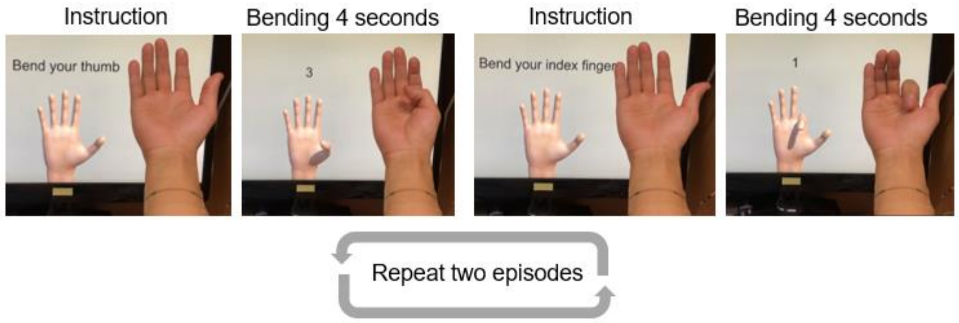

Supplementary Figure 12 | Snapshot of user following the instructions

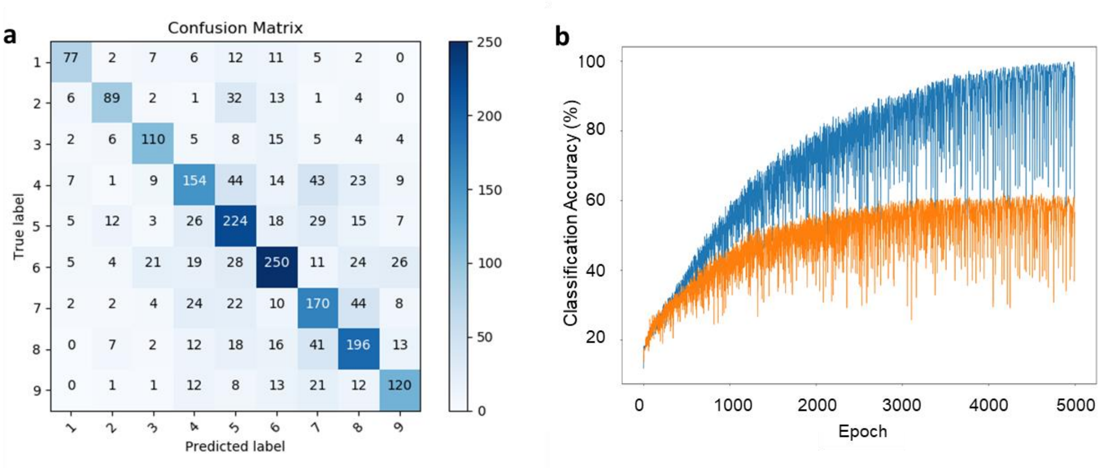

Supplementary Figure 13 | Keypad learning a. Confusion matrix of decoding the keypad typing. **b.** Classification accuracy of keypad input prediction.

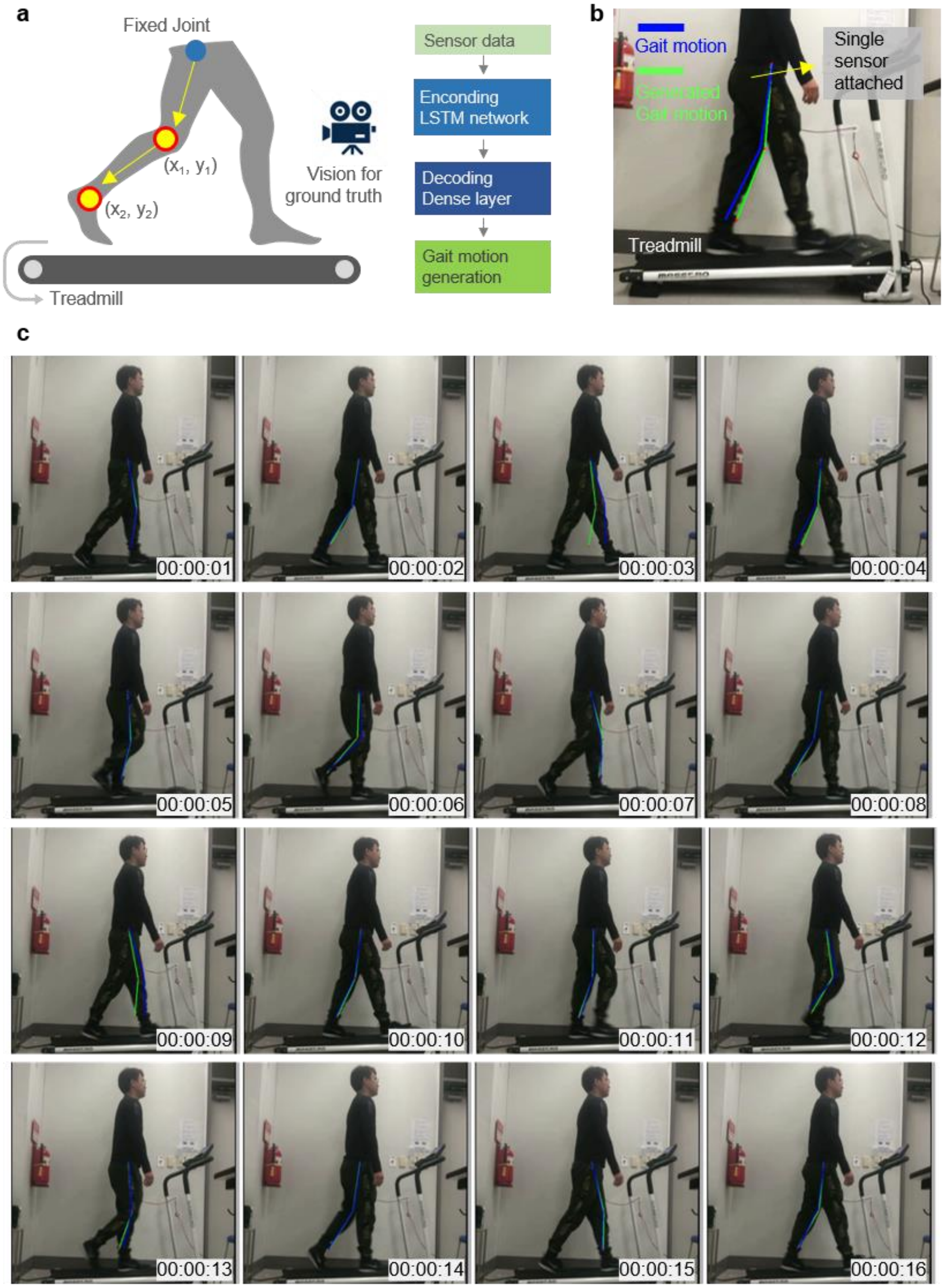

Supplementary Figure 14 | Predicting gait motions by a single sensor attached on pelvis a. Experimental settings of receiving gait signals. **b-c.** Successfully decoded gait motions.

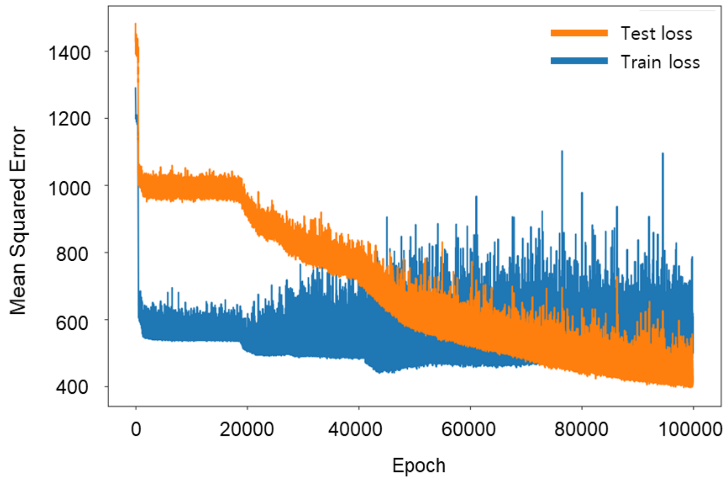

Supplementary Figure 15 | Mean Squared Error for gait motion prediction model

Supplementary Tables

Supplementary Table 1 | Comparison of the developed system in this study and the previous sensors

Type	Sensitivity	Stretchability	Necessary data rate	Sensing part	# of sensors	Dynamics motion	References
EMG	Need Amplifier	None	1000 Hz	8 finger gestures	128	None	Scientific Reports, 2016, 6, 36571
Resistive	GF 8.5	50 %	N/A	4 body states	1	None	Sensors, 2018, 18(9), 3109
EMG	Need Amplifier	None	1000 Hz	11 stroke shapes	6	None	Neurosci, 2016, 14
Resistive	GF ~2	<10 %	N/A	27 objects	1024	None	Nature, 2019, 569
Vision	N/A	N/A	N/A	Vision Camera	1	Available	Google Research 2019
EMG	Need Amplifier	None	1000 Hz	5 finger gestures	10	None	J Neuroeng Rehabil, 15, 57, 2018
Resistive This Study	**GF~2000 GF~300**	**<1 % <15 %**	**<20 Hz**	**5 Finger gestures**	**1**	**Available**	

Supplementary Table 2 | Sensor performance compared to previous sensors

Structure	Sensitivity	Stretchability	References
Intrinsically stretchable (ionic gel)	GF ~ 4 (0 ~ 800 %)<br>GF ~ 20 (800 ~ 1500 %)	1500 %	Small, 2019, 15, 1804651
Intrinsically stretchable (conductive fabric)	GF ~ 10 (0 ~ 250 %)<br>GF ~ 37 (250 ~ 500 %)	500 %	Adv. Mater, 2016,28, 6640-6648
Intrinsically stretchable (AuNW network)	GF ~ 7	50 %	Adv. Electron. Mater. 2015, 1, 1400063
SiNR serpentine	GF ~ 60<br>(depending on curvature)	15 %	Nat. Comm. 2014, 5747
Pt Crack structure	GF ~ 2000	2 %	Nature, 2014, 516, 222-226
Nanographene film	GF ~ 300	0.3 %	App. Phys. Lett. 2012, 101, 063112
**Crack + serpentine This Study**	GF ~ 3000<br>GF ~ 300	1 %<br>15 %	

Supplementary Note 1. Geometrical modeling of the quasi-static bending condition to exploit crack characteristics

The porous sintered region of the sensor under quasi-static bending condition should be cracked locally when local strain exceeds above critical crack strain, ε_c. The situation is schematically illustrated in **Supplementary Figure 1a**, the red zone depicts a cracked region whose local strain exceeds the critical crack strain, *dl* is linear displacement of bending stage, and l_{cp} is the projected length of cracked zone, and *L* is the initial length of the sensor. Since the first buckling mode of beam is sinusoidal[1], the sensor would bend along sinusoidal curve. Therefore, the shape of bent sensor can be modeled as,

$$w(x) = w_0 \cos\left(\frac{\pi x}{L - dl}\right) \tag{1}$$

The sensor attached to the substrate (PET, thickness = 10 μm) conformably, the local strain of upper face of cosine curve could be directly read as the sensor's local strain. The height of cosine is defined using geometrical restriction, constant length condition,

$$\int_0^{(L-dl)/2} w_0 \cos\left(\frac{\pi x}{L - dl}\right) \mathrm{d}x = \frac{L}{2} \tag{2}$$

Such a constriction yields the w_0 as the function of *dl*. From elemental calculus, we could derive the local strain of the sensor as the function of *x* and *dl*.

$$\varepsilon(x, dl) = \frac{h\pi^2 w_0(dl)\cos\left(\frac{\pi x}{L - dl}\right)}{2(L - dl)^2\left(1 + \left(\frac{\pi w_0(dl)}{L - dl}\sin\left(\frac{\pi x}{L - dl}\right)\right)^2\right)^{\frac{3}{2}}} \tag{3}$$

where *h* is the thickness of sample. The curvature of cosine which decreases monotonically along *x* direction leads same trend of its local strain. Therefore, we can divide the cosine curve in two sections;

the black zone: the local strain is lower than critical crack strain and the red zone: the local strain is higher than critical crack strain. (**Supplementary Figure 1a)**

The projected length of cracked zone divides the cracked and the non-cracked zone and is defined as following implicit form.

$$\varepsilon(l_{\mathrm{cp}}, dl) = \varepsilon_c \tag{4}$$

In a given critical crack strain, the projected length of cracked zone is function of *dl* and monotonically increases.

To observe the microscopic phenomena of cracking, we conducted a quasi-static bending experiment with simultaneously measuring the electrical resistance of the sensor. The modeled electrical resistance can be expressed using projected length of cracked zone and resistances per unit length, r_c, r_n. A resistance variation after cracking should be negligible since initial cracking process is occurred discontinuously (**Figure 3a**). When crack propagates beyond the initial void size slightly, the percolation of the electrical path could be broken. Such a change of physical parameter related to percolation probability around the percolation threshold significantly effects on resultant values[2] and we can treat each resistance as constant.

$$r(x) = \begin{cases} r_c \; for \; x|\varepsilon(x, dl) > \varepsilon_c \leftrightarrow x < l_{cp} \\ r_n \; for \; x|\varepsilon(x, dl) < \varepsilon_c \leftrightarrow x > l_{cp} \end{cases} \tag{5}$$

Therefore, the total resistance of the sensor is deduced as,

$$R(dl) = 2r_c l_c + r_n(L - 2l_c) \tag{6}$$

$$l_c = \int_0^{l_{\mathrm{cp}}} \sqrt{1 + w(x)^2} dx \quad (7)$$

where l_c is length of cracked zone. **Supplementary Equation 6** can be manipulated further

$$\frac{R}{R_0} = \frac{2}{L}(\alpha - 1)l_c + 1 \quad (8)$$

where $R_0 = r_n L$, initial resistance of the sensor, and $\alpha = r_c/r_n$, the resistance ratio between cracked and non-cracked zone. Combining **Supplementary equation 4, 7, and 8**, we successfully found the relationship of the linear deformation of bending stage and the sensor's resistance. There are only two free factors in the model, the critical crack strain, ε_c and the resistance ratio α. Our purpose of designing the quasi-static bending experiment was exploiting the characteristic of cracking process which is hidden behind the data. The model naturally provides the effective factor determining the sensor's performance, two free factors ε_c and α. In order to find the two characteristic factors corresponding the laser condition, we employed the least square regression to fit the experimental *dl vs.* R/R_0 data using ε_c and α. The data were well fitted; however, actual signal had some jump point inferring the discontinuous generation of micro cracks. The model stands on the assumption of continuous propagation of cracked zone.

Supplementary Note 2. The critical strain and propagated length of crack

When bending stress is applied to the as-prepared sensor, crack propagates from non-sintered to sintered region (**Supplementary Figure 1b**).

If a specific strain is applied to the conducting region, the crack in the non-sintered region will act as a crack seed in the sintered region, which will propagate the crack further. The relationship between crack depth and critical strain is essential to extract the performance characteristics of the sensor along with test in **Figure 3b**. According to Irwin[3], cracks are known to propagate under the following conditions.

$$G_c = -\frac{dU}{dA} = 2w_{\mathrm{f}} \tag{9}$$

where G_c is the critical energy release rate, U is potential energy of body, A is crack area, and w_{f} is fracture energy per unit area. Since total strain energy is potential energy plus work done by external stress, the potential energy of elastic body can be defined as follows.

$$U = S - W \tag{10}$$

Since the experiment illustrated at **Figure 3b** was conducted under quasi-static displacement equilibrium bending condition, work done by external force, W can be expressed by

$$W = Pl \tag{11}$$

Also, elastic strain energy S should be calculated by

$$S = \int_0^l P dx = \frac{Pl}{2} \tag{12}$$

where P is external force, and l is linear deformation. Therefore,

$$U = -\frac{Pl}{2} = -S \tag{13}$$

The energy release rate related to quasi-static displacement equilibrium condition is defined as follows.

$$G = \frac{1}{b}\left(\frac{dS}{dp}\right)_l = \frac{l}{2b}\left(\frac{dP}{dp}\right)_l \tag{14}$$

where b is thickness of the sensor, and p is the propagated length of crack. (**Supplementary Figure 1b**) We manipulated **Supplementary Equation 14** further for exploiting the relation between critical crack strain and crack depth. Considering the geometry of the sensor system, the energy release rate can be transformed,

$$G = \frac{L\varepsilon}{2b}\left(\frac{d(E\varepsilon wb)}{dp}\right)_l = \frac{EwL}{2}\left(\varepsilon\frac{d\varepsilon}{dp}\right)_l \tag{15}$$

where E is young's modulus of sintered region, w is width, and L is length of sintered region. Inserting the energy release rate (**Supplementary Equation 15**) to the critical condition occurring the cracking (**Supplementary Equation 9**), the following relation is satisfied.

$$\frac{EwL}{2}\left(\varepsilon\frac{d\varepsilon}{dp}\right)_{\varepsilon=\varepsilon_c} = 2w_\mathrm{f} \tag{16}$$

We integrated the **Supplementary Equation 16** both side with respect to p approximating that the bonding energy w_f is constant[4], and the initial strain-free state has no distinctive crack.

$$\varepsilon_c^2 = \frac{8w_\mathrm{f}}{EwL}p \tag{17}$$

Meanwhile, the fracture energy could be approximated by the atomic potential U_a with Taylor expansion about an equilibrium position

$$w_\mathrm{f} \sim \frac{1}{2\delta^2}\delta^2\left(\frac{\partial^2 U_a}{\partial r^2}\right)_{r=r_0} = \frac{1}{2}\left(\frac{\partial^2 U_a}{\partial r^2}\right)_{r=r_0} \tag{18}$$

where δ is displacement to occur a cracking of body, r_0 is equilibrium position of atom, and r is elongation coordinate. Young's modulus can be also approximated by similar way,

$$E = \frac{dP}{dr}\frac{L}{wb} \sim \left(\frac{\partial^2 U_a}{\partial r^2}\right)_{r=r_0}\frac{L}{wb} \tag{19}$$

With above ingredients, we have the approximated relation between critical crack strain and crack depth for the tiny strain regime of a thin film,

$$\varepsilon_c^2 \sim \frac{4b}{L^2}p \tag{20}$$

The scaling comparison leads the order of magnitude of the critical crack strain using the sintered region's geometry, $p \sim O(10^{-7}\mathrm{m}), b \sim O(10^{-6}\mathrm{m}), \text{and } L \sim O(10^{-3}\mathrm{m})$.

$$\varepsilon_c \sim O\left(\frac{10^{-6}10^{-7}}{10^{-6}}\right)^{\frac{1}{2}} = O(10^{-3}{\sim}10^{-4}) \tag{21}$$

We could confirm the validity of **Supplementary Equation 20** through the result indicated **Figure 3c**. The characteristics of cracking were calculated by the method found in **Supplementary Note 1**; $\varepsilon_c =$

$2.977 \times 10^{-4}, \alpha = 1.846$ for 9 mW, $\varepsilon_c = 2.40 \times 10^{-4}, \alpha = 1.4$ for 11 mW. The order of magnitude of the critical strain ε_c is 10^{-4} for each case which is good agreement with **Supplementary Equation 21**. Furthermore, if we assumed that the resistance ratio α is linearly proportional to the length of crack,

$$\left.\frac{\varepsilon_c^2}{\alpha}\right]_{\text{power}=11\text{ mW}} = 4.80 \times 10^{-8} \sim \left.\frac{\varepsilon_c^2}{\alpha}\right]_{\text{power}=9\text{ mW}} = 4.11 \times 10^{-8} \qquad (22)$$

Since we fabricated the sensors maintaining the same geometry, above quantity should be similar for varying the laser power. Note that the independent approach to investigate the crack characteristics merged in **Supplementary Equation 4 and 20**.

Supplementary Note 3. The relation between crack asperity and the sensitivity

Kang *et. al.*[5] found that the crack asperity distribution has fractal self-similarity to the grain size distribution by renormalization group theory. They brought the log-normal distribution which is well explained grain size distribution. The key fitting parameters are the grain size parameter ε_0 and the deviation μ. Defining the log-normal distribution as crack asperity distribution, they derived the normalized conductance S with respect to the strain ε.

$$S = \frac{1}{2}(1 - \mathrm{erf}\left(\frac{\ln(\varepsilon/\varepsilon_0)}{\mu}\right)) \tag{23}$$

where erf(x) is the error function, ε_0 is grain size parameter, and μ is the deviation of distribution. As shown in **Supplementary Figure 2**, the sensor data is fitted by Equation 23, and found the large grained structure has higher sensitivity. (ε_0 = 0.2489, $\mu = 1.196$ for 6 mW, ε_0 = 0.38, $\mu = 1$ for 9 mW). Note that the actual grain size ($d_0 = k\varepsilon_0$) cannot be directly defined by the grain size parameter (ε_0), since the parameter k differs by the laser power.

Supplementary Note 4. Modulus of the particle layer

Modulus of the pristine and the sintered particle layer is measured by nanoindentation testing (Ultra precision surface Mechanical Analyzer, Anton Paar). The presented result in **Supplementary Figure 4a** correspond to an average of 7 indents. Sintered particle layer is showing a lower penetration depth at the same force. Pressure mark made above particle is shown in **Supplementary Figure 4b**. Indentation modulus of the pristine particle layer is calculated as 1.246 GPa, whereas the sintered particle is 2.024 GPa.

Supplementary Note 5. Mechanical properties of the serpentine structure

As shown in **Supplementary Figure 5**, various experiments evaluating the mechanical properties of the sensor is conducted. Since the serpentine pattern has higher conducting path than the single lined sensor, the output performance is much more stable as depicted in **Supplementary Figure 5a**. The initial cracking is done at 15% as shown in **Supplementary Figure 5b**. The dynamic cycle response is shown in **Supplementary Figure 5c**. The shape parameter of the structure is illustrated in **Supplementary Figure 5d**.

Supplementary Note 6. Data Processing and Network Design

Two datasets were used to train the model: a dataset for pre-training and a dataset for recalibration. The data values within the pre-training dataset (15930 frames) range from 170 to 195 (units). 3,186 frames were collected from 100 seconds of finger motion data for each finger. The recalibration dataset contains 1,000 data frames, or 200 data frames from 8 seconds of data for each finger. The data values within the recalibration dataset exhibit a different range, which is dependent on the position of the sensor, the user, and the sensor itself.

By using a sliding time window of size 16 along the data sequence, 16 frames of consecutive sensor values were regarded as a single input. This was done to utilize the temporal behaviors of the sensor signals. Each input was labeled with two float values, r and θ ($0 \leq r, \theta \leq 1$). θ represents the finger with which the movement is done. θ values start from 0 if the movement is from the thumb and ends at 1 if the movement is from the little finger, with the values for the fingers in between increasing linearly by 0.25.

For r, which represents how bent the finger is, we picked the local maximum and minimum of the sensor values to distinguish the bent and unbent states. Each local maximum and minimum were labeled as having an r value of 1 and 0, respectively. For intermediate sensor values, the r values were linearly interpolated as the ratio of the difference between the current sensor value and the closest local minimum and the difference between the closest local maximum and the closest local minimum.

Although we could have used a motion capture device or depth camera to be more precise with our r values, we decided to avoid such devices as we want a simple and convenient method to train our model with only our single-channeled sensors.

We then split the pre-training dataset into training and test subsets. By chronologically organizing and splitting the 16-frame-long inputs belonging to one data sequence into 10 consecutive groups of equal size and randomly choosing eight of those groups for the training set and two for the test set, we increased the regularization effect by minimizing the number of frames that appear in both the training

set and the test set. This was done for all data sequences in the pre-training dataset. In both the training dataset and the test dataset, the same number of groups were selected for each finger to even out the data distribution.

Our network consists of a 5-layered LSTM network, a type of Recurrent neural network and two separate 3-layered dense networks. Recurrent neural network is a type of neural network typically designed for dealing with sequences of inputs. RNN is composed of RNN units which combine current input and hidden vector passed from previous unit to generate current output. Therefore, RNN is well-suited to processing time series data. Unlike standard RNNs, LSTM networks additionally train three gates (input gate, output gate, and a forget gate) to regulate the flow of information from one cell to another. The overall structure of the LSTM is illustrated in **Supplementary Figure 9.**

LSTM unit takes an additional vector, c_{t-1}, the previous memory cell. Following its literal meaning, memory cell contains integrated information from previous LSTM units. LSTM has a forget gate, an input gate, an output gate inside each unit controlling the next memory cell, c_t, to be passed on to the next unit. Two activation functions, σ_g, for sigmoid function and $\tanh$ for hyperbolic tangent functions, are applied for each gate outputs to control the range of output vectors. Here's the overall equation of three gates vectors.

$$f_t = \sigma_g (W_{xf} x_t + W_{hf} h_{t-1} + b_f) \quad (24)$$

$$i_t = \sigma_g (W_{xi} x_t + W_{hi} h_{t-1} + b_i) \quad (25)$$

$$o_t = \sigma_g (W_{xo} x_t + W_{ho} h_{t-1} + b_o) \quad (26)$$

Three vectors, f_t, i_t, o_t, are parameterized by its corresponding weights matrices W. Weight matrices are trained so that the unit can modify memory cell based on current input and hidden vector. Taking current input vector, x_t, and hidden vector, h_{t-1}, as input, gates generate vectors ranging from 0 to 1. Sigmoid function, σ_g, is used to bind the gates vectors in between 0 and 1. However, they are multiplied to different vectors to achieve different purposes.

$$s_t = \tanh(W_{xg}x_t + W_{hg}h_{t-1} + b_g) \quad (27)$$

$$C_t = f_t \odot C_{t-1} + i_t \odot s_t \quad (28)$$

$$h_t = o_t \odot \tanh(C_t) \quad (29)$$

Following its literal meaning, f_t is multiplied element-wise to previous memory cell C_{t-1} to determine how much information from the pass are going to be forgotten in current unit. Symbol $\odot$ means Hadamard product, which also stands for element-wise product. Input gate vector i_t, in contrast, determines the amount of current input x_t and h_{t-1} to be taken account in current memory cell C_t. Input gate vector, i_t, is multiplied by s_t and added to memory cell. s_t represents memory generated from current input and previous hidden state. Hyperbolic tangent is used to generate s_t so that not only the magnitude but also the sign of s_t is considered. Finally, Output gate vector is multiplied by the current memory state C_t to generate a new hidden state h_t and it is passed to the next state. The last LSTM unit will take a memory cell containing key information summarizing passed input sequence and generate output based on it. Generated latent output vectors imply sensor patterns for sensor signals within a time window.

Since we aimed to not only accurately determine the hand motion but also quickly re-calibrate the sensor when needed, we determined the number of layers for each network by comparing testing accuracy versus training time as shown in **Supplementary Figure 10**. A 5-layered LSTM network achieved the fastest training time to reach the same level of accuracy. However, 9-layered network shows significantly higher loss values than other shallower networks. While a deeper neural network can interpret more complex patterned data, it can also easily be overfitted for a bounded dataset. 9-layered network contains an excessive number of parameters so that it is too biased to training dataset. As a result, the network is overfitted to the training dataset so that it is not generalized to predict unseen data pattern. In particular, we are utilizing a sequence of a single sensor value to generate corresponding hand motion. Thus, our task has a relatively low data dimension. Furthermore, the data is collected manually by attaching it on human arms, making it difficult for us to collect huge amount of data. Thus, the LSTM with 3 to 7 layers were more suitable for our current dataset size, while 9-layered LSTM was too deep. This result can be changed if we collect more data from more people.

Each frame within a 16-frame-long input was sequentially passed into LSTM units to produce a 128-dimensional hidden vector. Three trainable gates in each LSTM unit controlled the information flow from a unit to the next unit, preventing the gradient vanishing problem and enriching the information received by dense layers. While LSTM layers could process sequential data inputs to generate latent vectors summarizing temporal behaviors of sensor signals, we are aiming to map such high dimensional latent vectors into our metric space expressing single-finger motions. Therefore, a decoding network that maps implicative latent vectors into coordinates in half-circle metric space is needed. Decoding network is composed of two separate dense layers. Dense layers allow our model to decide the dimension of output vectors while decoding information embedded in latent vectors from the encoding network.

The resulting 128-dimensional vector was concatenated with the input to create a 144-dimensional vector, which is then passed onto the decoding network consists of two separate dense layers groups. One generates r values while the other generates θ values. The rectified linear unit (ReLU) was used as the activation functions for the dense layers. To prevent overfitting to the small recalibration training dataset, 30% dropout was applied to all layers. We implemented the network using the PyTorch deep learning framework. The Adam optimizer of learning rate 10^-4 was used for training the network. The Euclidean distance between the predicted point and the labeled point within our metric system was calculated and used as the loss function for training our network.

To visualize the user's hand motion for our real-time demo, a virtually generated hand that mirrors the user's hand motion was generated using the cross-platform visual engine Unity (unity.com). We modified a VR hand motion asset in the Unity Asset Store to implement our demo. After a user attaches the sensor, our network generates r and θ values, which are then sent to our Unity application through socket connections and used to move the virtual hand in accordance with the values and the corresponding hand motion on the metric space we defined in **Figure 4b**. Socket connections were created with the Python socket module API. For points labeled with a θ value that is not a multiple of 0.25, we projected the point to the nearest finger and moved the corresponding finger of the virtual hand.

To avoid collisions caused by simultaneous hand motion orders, fixed time step that determines when physics calculations are performed in Unity is set as 0.3 seconds.

Supplementary Note 7. Keyboard typing

In addition, we collected sensor signals while typing number pad keyboard to demonstrate the use cases where wrist movements and finger movements are coupled. We collected 12000 sensor frames while typing a number keyboard (**Supplementary Figure 13**). We again grouped 16 consecutive sensor signals into one input. Each input is labeled from 1 to 9 that are pressed by fingers. Therefore, our decoding layer is now transformed to generate 9-dimensional vector representing likelihoods of each 9 classes.

Supplementary Note 8. Predicting the gait motions

To verify the generalizability of our sensor, we also checked whether our model can generate the gait motions of a user using data from a sensor attached to the left side of their pelvis. By recording a 1920 x 1080 resolution video of the user's gait motion while gathering sensor signals, we collected 3145 frames of video data and 5158 points of sensor data.

Each frame was manually labeled with the pixel coordinates of the pelvis, left knee and left ankle, and then synchronized with the sensor values obtained during the frame (**Supplementary Figure 14a**). The labelled position of the pelvis was fixed for all frames to clearly show gait motion between frames. For sensor values collected between two consecutive video frames, the coordinates of the left knee and ankle were estimated through linear interpolation of their coordinates in the two frames. Just as we preprocessed the data for hand motion generation, we grouped 16 consecutive sensor signals as one input so that our model can utilize the sequential patterns of the sensor signals, with each input labelled with the corresponding gait motion of the last signal within the input.

To generate positions of the ankle and the knee, we modified the last layer of our decoding network to generate a 4-dimensional output vector instead. With 3 dense layers and dropout combined, the decoding network is transformed so that it maps a latent vector generated by the encoding network to two points within the image space of the video. Thus, the first two values of the output vector represent the x and y coordinates of the left knee, while the last two values represent the coordinates of the left ankle. The loss function for our model training (**Supplementary Figure 15**) is the mean squared errors between the labeled points and the predicted points. 80% of the sensor frames are used as the training set and the remaining 20% of the sensor frames are used as the testing set. The results of predicting gait motions form the test sets are demonstrated in **Supplementary Figure 14b-c.**

Supplementary References

1. Archer, R. R. et al. An introduction to Mechanics of Solids. *Tata McGraw-Hill Education* (2012).

2. Shante, V. K. et al. An introduction to percolation theory. *Adv. Phys.* **20**, 325-357 (1971).

3. Irwin, G. R. Onset of fast crack propagation in high strength steel and aluminum alloys. *In Sagamore Research Conference Proceedings.* **2**, 289-305 (1956).

4. Anderson, T. L et al. Fracture mechanics: fundamentals and applications. *New York: CRC press,* (2005).

5. Kang, D. et al. Ultrasensitive mechanical crack-based sensor inspired by the spider sensory system. *Nature* **516**, 222-226 (2014).
