# Supplementary material for: A deep-learned skin sensor decoding the epicentral human motions
Source: Nat Commun. 2020 May 1;11:2149. doi: 10.1038/s41467-020-16040-y (PMC7195472; doi:10.1038/s41467-020-16040-y)
Supplement: Supplementary file 2 — Description of Additional Supplementary Files [file 41467_2020_16040_MOESM2_ESM.pdf]

Description of Additional Supplementary Files

Supplementary Movie 1. Decoding the dynamic finger motions
